# Performance of Plain Concrete and Cement Blocks with Cement Partially Replaced by Cement Kiln Dust

**DOI:** 10.3390/ma14195647

**Published:** 2021-09-28

**Authors:** Yasir M. Alharthi, Ahmed S. Elamary, Waleed Abo-El-Wafa

**Affiliations:** 1 Civil Engineering Department, College of Engineering, Taif University, P.O. Box 11099, Taif 21944, Saudi Arabia; y.harthi@tu.edu.sa; 2 Civil Engineering Department, Assiut University, Assiut 71515, Egypt; wam@aun.edu.eg

**Keywords:** cement kiln dust, compressive strength, tensile strength, concrete air content, cement blocks

## Abstract

The growth of the construction industry has led to the greater consumption of natural resources, which has a direct or indirect negative impact on the environment. To mitigate this, recycled or waste materials are being used as a partial substitute in the manufacture of concrete. Among these waste materials is cement kiln dust (CKD), which is produced during cement production. This study investigated the potential benefits of replacing part of the cement with CKD in two construction applications, i.e., plain concrete and cement blocks. This reflects positively on cost, energy, and the environment, since putting CKD in a landfill damages agricultural soil and plant respiration. In this study, an experimental program was carried out to study how replacing various percentages of ordinary portland cement (OPC) with CKD affected the compressive strengths, the tensile strengths, and the air contents of concrete and cement blocks. Although the results showed that the compressive and tensile strengths decreased as the amount of CKD increased, the air content of the concrete increased, which showed that 5% CKD was suitable for such applications. The results were used to propose two equations that approximate the concrete and cement block compressive strengths according to the CKD replacement percentage.

## 1. Introduction

Over the past few decades, the demand for construction materials has increased due to the worldwide growth of construction projects. Unfortunately, this demand is fulfilled by the overconsumption of natural resources endangering the environment [1]. Accordingly, researchers are motivated to reduce the impact of construction materials on the environment and enhance the mechanical properties of construction materials. This has led to the use of waste materials (recycled aggregate and rubber) and cement kiln dust (CKD) in the manufacture of concrete and studies on how these materials affect its behavior [2,3,4,5,6,7].

Małek et al. [2] investigated the use of ferronickel slag waste aggregate (FNSWA) in place of granite aggregate and found an increase in compressive and flexural strength of 31% and 66%, respectively. Padmini et al. [3] studied the influence of parent concrete on the properties of recycled aggregate concrete. This study showed that achieving a desired compressive strength using recycled aggregate concrete requires using a lower water–cement ratio and a higher cement content than for concrete with fresh granite aggregate.

Experimentally, Chalangaran et al. [4] improved environmental noise absorption after replacing sand aggregate with recycled rubber crumbs. Fifteen percentages of fine- and coarse-grained crumbs substitutions improved sound transmission losses by 190% and 228%, respectively. They also reported [7] that the ultimate compressive strength, the tensile strength, and the modulus of elasticity of the concrete with rubber crumbs are reduced. The negative impact of the rubber crumbs was reduced by using an optimum percentage of nanosilica and metakaolin additives.

One of waste materials that researchers have used in concrete is CKD, a fine powdery material resulting from cement manufacture. Studies showed that the greatest health danger in cement production comes from dust, and several studies have been conducted by references [8,9,10,11] to assess the influence of cement dust in the workplace and how it stimulates abiotic stress responses in plants.

The environmental concerns related to portland cement (PC) production and emission and the disposal of CKD are becoming progressively significant, because 80% of CKD ends up in landfills. The extreme contamination of the surrounding environment highlights the need to find more environmentally friendly ways of disposal.

Using CKD as a partial replacement in PC is currently fraught with difficulties, and many of its applications continue to be investigated, for example, as a component in cement, an agricultural fertilizer, a soil and wastewater stabilizer, a partial replacement of soda in glass production, an antistripping agent in asphalt, and a subgrade for highway construction as stated by Shervan [12].

Maslehuddin et al. [13,14] studied properties of blended cement concretes (CKD). The mechanical properties of CKD concrete specimens were assessed using drying shrinkage and compressive strength, while the durability was assessed using electrical resistivity and chloride permeability. The compressive strength and the drying shrinkage strain of the concrete samples decreased, as the quantity of CKD increased. The chloride permeability improved, and the electrical resistance decreased when CKD was added. Finally, it is recommended that the amount of CKD in concrete be limited to 5%.

Ravindrarajah et al. [15] studied CKD as a partial replacement for cement in concrete and found that it is a cementitious substance that delays the cement setting, increases water consumption to achieve consistency and reduces the concrete strength. El-Sayed et al. [16] and Batis et al. [17] examined the impact of CKD on the cement paste compressive strength and the embedded reinforcement corrosion behavior and found that up to 5% CKD replacement by weight has no negative impact on the cement paste strength or reinforcing passivity. In addition, Batis et al. [17] reported that adding CKD and blast furnace slag (BFS) in a correct amount increases the compressive strength and the corrosion resistance of PC.

Abo-El-Nien et al. [18] studied the partial replacement of BFS cement with CKD and the effects of kiln meal and CKD on the PC paste strength. Compared to free cement, the findings revealed a high degree of hydration and a reduced compressive strength. Shoaib et al. [19] studied the effect of replacing ordinary PC and BFS cement with CKD on the splitting tensile strength of concrete mixes at various ages (1, 3, and 6 months) and concluded that it dropped as the proportion of CKD increases and it was minimal at 10% CKD.

Al-Harthy et al. [20] investigated the effect of CKD on the toughness and flexure strength of concrete mixes 3, 7, and 28 days after casting. They discovered that the mixes containing less than 5% CKD had similar toughness and flexural strength values to the control mix, especially at the water-to-binder ratio of 0.50. Mosleh et al. [21] investigated the strong growth of PC pastes with the addition of kiln meal and kiln dust, finding a higher degree of hydration and a lower compressive strength than when free cement was added.

Heikal et al. [22] studied PC clinker, BFS, and CKD composites. Three mixtures of slag cement were prepared, each mixed with 2.5%, 5.0%, 7.50%, and 10.0% CKD. The authors found that the partial replacement of BFS with CKD improves PC clinker’s setting periods, electrical conductivity, and fluidity. Udoeyo and Ridnap [23] studied the characteristics of hollow sandcrete blocks with added CKD as a substitute for ordinary portland cement (OPC) and concluded that when CKD is used to replace cement, the compressive strength and density of the blocks fall as the proportion of CKD replacement rises, but the percentage of water absorption of the blocks increases.

## 2. Research Goal and Methodology

This research effort was designed to open new industrial areas for recycled CKD. Two main construction applications were studied in this experimental work program. The first was the effect of CKD replacement on plain concrete strength and air content. In this application, three different groups were investigated. The first group consisted of concrete samples tested for concrete compressive strength after being prepared with 0%, 2%, 5%, 8%, 10%, 15%, and 20% CKD replacements. The second group consisted of concrete samples prepared with 0% and 5% CKD replacements and tested for tensile strength. To test the effect on air content, the third group included concrete samples prepared with 0%, 5%, 10%, 15%, and 20% CKD replacements.

The second construction application was related to hollow cement blocks. Cement block specimens were prepared with 0%, 10%, 15%, 20%, and 25% CKD replacements and tested to study their effect on compressive strength and water absorption. The values are represented in the database to form equations that reflect the impact of the CKD% on the compressive strength.

## 3. Experimental Work

An experimental program was conducted to study the influence of replacing a percentage of OPC with CKD in 120 specimens divided into two groups: the specimens of the first group were used to measure the most important parameters of concrete, and those of the second group were used to scale the effect on cement blocks. The amount of CKD in the mix was expressed as a percentage of the total weight of cement in the mix. The types of test and specimen numbers and details are listed in Table 1. This experimental program conducted five different tests: three for plain concrete and two for the cement blocks.

### CKD Materials

The materials used in this research were untreated raw CKD collected from electrostatic precipitators and OPC products from a Madina cement factory (Madina, KSA). A comparison between the chemical compositions of CKD produced from the Madina cement factory with the CKD limits standard in the UK is illustrated in Table 2.

The percentage of sulfate, as well as alkalinity, chloride, and silica concentrations, was within acceptable limits, according to this analysis. On the other hand, the chemical composition of cement varied based on raw materials and the manufacturing processes used. The quantity of decarburization of the calcium carbonate in the clinker-making raw materials was reflected in the level and variability of the loss on igniting the CKD.

## 4. Procedures and Result Discussion

### 4.1. Concrete

The concrete mixture can be designed in a number of ways, but the British Standard (BS) mix was used in this study since it is concerned with cube compressive strength. Using the coefficient of variation and the lowest strength as a percentage of the mean strength, the BS technique defined the characteristic strength. This approach also took into account the influence of the fine aggregate on the determination of the fine-to-total aggregate content in the mix by ensuring that the aggregate grading followed the grading zones.

The approach, suitable for OPC (type I), uses a hypothetical concrete mix with a moderate cement content (a water/cement (*w*/*c*) ratio of 0.5) that is thoroughly compacted, correctly cured and cast with various types of cement and coarse aggregate and tested at various ages. The specified quantities are sufficient just for plain concrete.

The fine and coarse aggregates used in the mix were graded using sieve analysis in accordance with BS 882:1983. The coarse aggregate size varied from 20 to 5 mm sieve size, while the fine aggregate size ranged from 5 to 0.61 mm. The *w*/*c* ratio (0.48), the cementitious material content (250 kg/m^3^), and the fine/total aggregate ratio (0.33) were all components. The relative density and the bulk density of the aggregate were measured and found to be 2.65 and 1645 kg/m^3^ for coarse and 2.70 and 1750 kg/m^3^ for fine aggregate.

#### 4.1.1. Effect of CKD on Concrete Compressive Strength

The design, treatment, and control conditions of the concrete mixes, with and without CKD, were the same according to BS 1881-108 [24]. To ensure uniformity, the ingredients were combined in a dry condition for roughly one minute. Water was progressively added to the mixture, which was then blended with workability admixtures. For an additional two minutes, the materials were mechanically combined.

A traditional slump test was used to determine the consistency of fresh concrete and the temperature. Specimens of a 150 mm cube were prepared from each concrete mix to be tested in compression after 28 days. During the concrete placement, a vibrating table was used to verify that the concrete was fully compacted. After 24 h, the cube specimens were demolded and submerged in water, until they were examined. Six cubes (150 mm × 150 mm × 150 mm) were cast from each mix and cured in clean water for 28 days. Compression tests were performed on standard cubes according to BS 1881-116 [25].

**Figure 1:**
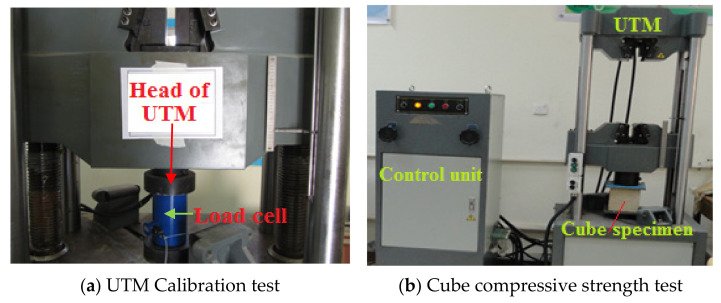
Concrete cubes after curing and during the test: (**a**) UTM (Universal Testing Machine) calibration test; (**b**) cube compressive strength test.

The crushing load was measured using a Universal Hydraulic Testing Machine with a capacity of 2000 kN to evaluate the compressive strength of a concrete sample as illustrated in Figure 1. In this part of the study, 42 cubes were tested to examine the replacement effect of CKD on the concrete compressive strength. The proportion of cement replacement by CKD was the most important component in this experiment. Seven samples were prepared and tested with 0% (control), 2%, 5%, 8%, 10%, 15%, and 20% CKD.

**Figure 2:**
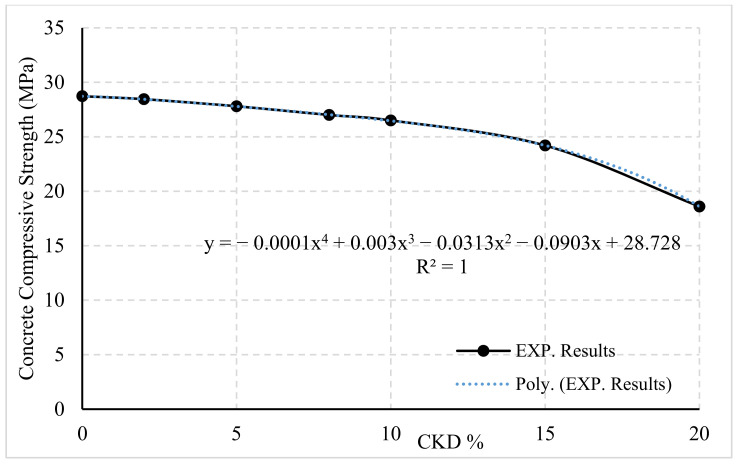
Compressive strength versus percentage of CKD.

The compressive strength results found experimentally for the 42 cubes were averaged and are listed in Table 3. The relationship between the average compressive strength and the replacement percentage of CKD is shown in Figure 2. A nonlinear regression analysis was used as recommended by [26]. A fourth-degree formula was fitted and created using a numerical database, as proposed in Equation (1). The curve plotted in Figure 2 reflected the effect of CKD on the concrete compressive strength after 28 days. Equation (1) was proposed to predict the compressive strength of a concrete mix that uses CKD as a partial replacement for cement:

(1) $$f_{\mathrm{cu}}=-1\times E^{-4}\times K^{4}+0.003\times K^{3}-0.0313\times K^{2}-0.0903\times K+F_{\mathrm{cu}},$$

where K is the percentage of CKD;$\;F_{\mathrm{cu}}$ is the designed concrete compressive strength with 0% CKD; and $f_{\mathrm{cu}}$ is the anticipated concrete compressive strength after using K percentage of CKD.

The CKD describes fine-grained, extremely alkaline particulate material mostly made of oxidized, anhydrous, micron-sized particles. The bulk of prior research has found that fine CKD particles have greater sulfate and alkali concentrations and less lime content [27]. As a result, references [28] and [29] concluded that the high alkali content is responsible for the loss of compressive strength.

#### 4.1.2. Effect of CKD on Concrete Tensile Strength

This study was performed to investigate the influence of replacing cement with CKD up to 5% on concrete tensile strength. Concrete mixes with and without CKD were mixed, treated and controlled under the same conditions. The components were combined in a dry condition for about a minute. For a further two minutes, all of the ingredients were mechanically combined to guarantee the homogeneity of the mix.

**Figure 3:**
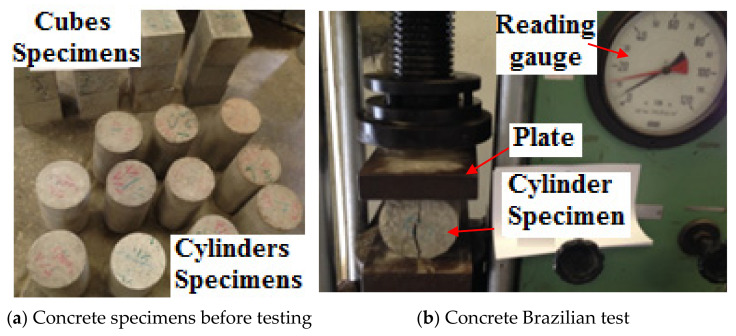
Cylinder specimen after curing and testing: (**a**) concrete specimens before testing; (**b**) concrete Brazilian test.

The conventional slump test measured the consistency of fresh concrete. Standard cylinders with a diameter of 150 mm and a height of 300 mm were prepared from each concrete mix according to ASTM C31 [30], as shown in Figure 3a. The ASTM C496 [31] test for splitting the tensile strength of cylindrical concrete specimens standard was used to conduct the splitting tensile test on the prepared standard cylinders.

All the molds were placed on a vibrating table while placing the concrete to ensure full compaction, and 6 cylinders were cast from each mix. After 48 h, all cylinder specimens were demolded and immediately submerged in potable water for 28 days. As shown in Figure 3b, all specimens were crushed in a testing machine. During the test, the load was monitored to determine the samples’ tensile strengths.

In this study, a total of 12 cylinders were tested to investigate their tensile strengths after replacing cement with CKD. The main factor considered in this study was the percentage of CKD replacement (0% and 5%). The results are listed in Table 4 and showed no significant difference in strength (approximately 3%).

#### 4.1.3. Effect of CKD on Air Content

**Figure 4:**
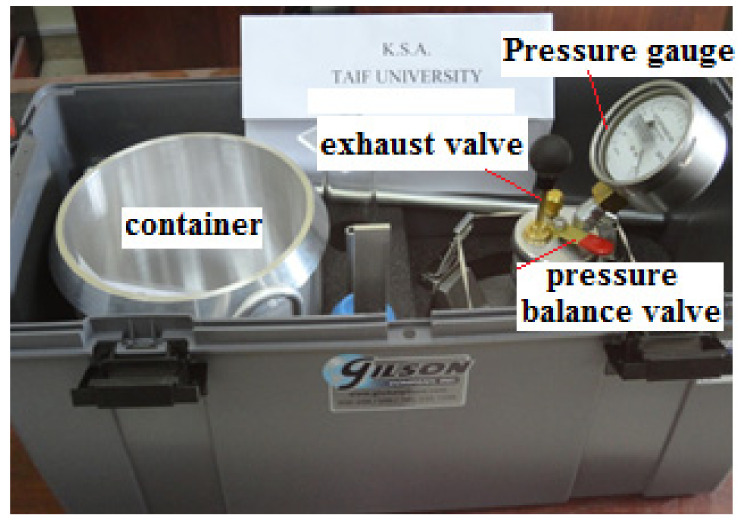
Air content testing apparatus.

The gross air contents of 15 concrete specimens were determined using the same mix that was previously used to determine compressive and tensile strength. The tests were performed using the pressurized technique according to ASTM C231 [32] for five different percentages of CKD (0% (control), 5%, 10%, 15%, and 20%). Figure 4 shows the Gilson equipment that was used to measure air content.

**Figure 5:**
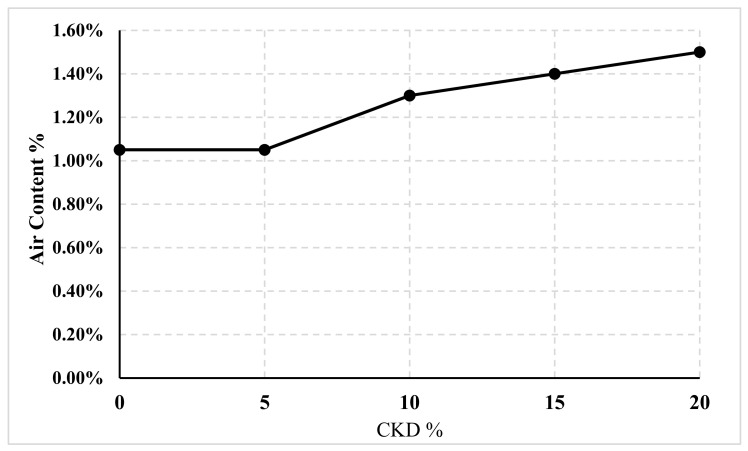
Relation between air content and CKD percentage.

The results are recorded in Table 5 and plotted in Figure 5.

Table 5 presents five groups of samples, each having three specimens as listed in column 1. Column 2 shows the percentage of CKD for each group, column 3 shows the recoded air content for each specimen, and column 4 shows the average results for each group.

These data showed that up to 5% CKD replacement can be ignored. When it was increased to 10%, the air content increased by 24%. In the last two cases, i.e., 15% and 20% CKD replacements, the air content rose by 33% and 43%, respectively, compared to the control mix.

#### 4.1.4. Water Cement Ratio and CKD Replacement Effects

The results of this investigation for the concrete compressive strength effect were in accordance with the results of a previous study [15,33]. According to reference [15], for the control and the modified concrete, the relationship between compressive strength (f) at 28 days and the ratio of the total volume of the cementitious material to the volume of water (R) was given by F = 125.6R − 39 (R unit: *N*/mm^2^). The expression for R is also given by R = (V_c_ + kV_d_)/V_w_, where V_c_, V_d_, and V_w_ are the volumes of cement, kiln dust, and water, respectively, and k is the cementing efficiency factor and written as:

k=CRQDQwcNwcDM−1,

where $C_{RQ}$ is the weight of cement (kg/m^3^); $D_{Q}$ is the weight of CKD (kg/m^3^); wcN is the water/cement ratio determined from the plotted curve between strength and *w*/*c*; and wcDM is the water/cement ratio in a design mix with 0% CKD.

To obtain *k*, wcN was calculated for different concrete strengths of the control mixtures with different *w*/*c* ratios, and the strengths after 28 days were plotted to derive the strength and *w*/*c* relationship curve [33]. The resulting curve can be used to obtain the effective *w*/*c* ratios for mixtures with different CKD percentages.

### 4.2. Hollow Cement Blocks

#### 4.2.1. Effect of CKD on Cement Block Compressive Strength

**Figure 6:**
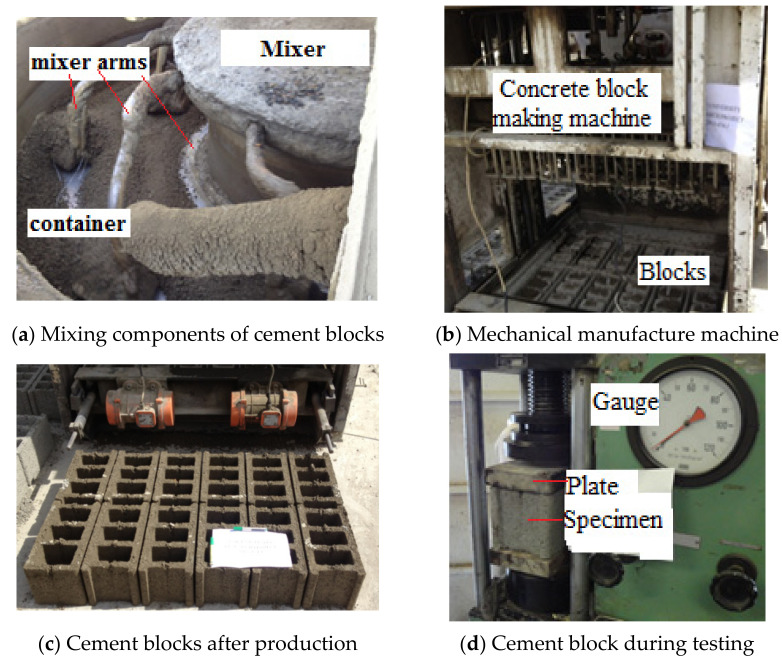
Hollow blocks, and stages of manufacture and under testing: (**a**) mixing components of cement blocks; (**b**) mechanical manufacture machine; (**c**) cement blocks after production; (**d**) cement block during testing.

The study was conducted according to ASTM C140 [34]. Twelve hollow cement blocks (200 mm × 200 mm × 400 mm) were manufactured using mix components 1 cement: 1.66 sand and 3.5 coarse aggregate. The quantities required to produce one block were 32 *N* cement: 53.5 *N* sand: 113 *N* coarse aggregate. To reach a uniform mixture, the ingredients were mixed in a dry state for about three minutes, after which water was gradually added. The blocks were mixed and formed mechanically as shown in Figure 6a–c.

In this part of the investigation, four different percentages of CKD were used: 10%, 15%, 20%, and 25%. The blocks with and without CKD were treated, controlled and cured under the same conditions until compression testing after 28 days, as shown in Figure 6d.

The results of the compression test are listed in Table 6. In each case, six blocks were tested, and the average compressive strength was determined. The gross and net areas were 74,600 and 44,225 mm^2^, respectively.

**Figure 7:**
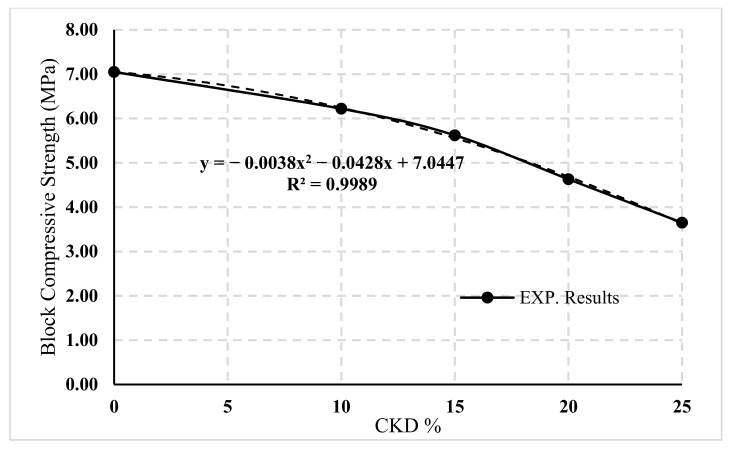
Relation between the block compressive strength and the CKD percentage.

Figure 7 shows the relation between the compressive strengths corresponding to gross area and CKD percentages.

By applying the regression analysis reported by reference [26] to the experimentally obtained numerical database, a nonlinear regression model based on a second-degree formula was fitted for this curve. Equation (2) was proposed to predict the compressive strength of blocks for the partial CKD replacement:

(2) $$f_{\mathrm{cu}-b}=-0.0038\times K^{2}-0.0428\times K+F_{\mathrm{cu}-b},$$

where K is the CKD percentage in the cement mix; $F_{\mathrm{cu}-b\;}$ is the block compressive strength with 0% CKD; and $f_{\mathrm{cu}-b}$ is the anticipated block compressive strength after K percentage of CKD.

#### 4.2.2. Effect of CKD on Cement Block Absorption

The present study was concerned with an analysis of the percentage of water absorption of hollow cement blocks (200 mm × 200 mm × 400 mm) with and without CKD conducted with respect to ASTM C140 [34].

The sequence of block formation was performed using the same procedure described in the previous section. The blocks were kept in an electronic oven under 100 °C for 24 h, weighed, subjected to the same drying conditions in the oven for half an hour and then reweighed to ensure that the weight did not change. The blocks were immersed in water for 24 h and then weighed at the end of that time.

The results from both stages are shown in Table 7, which showed the average weight at the end of the dry stage and the absorbed water. The percentage of water absorption in the control specimen was 4.27%, and this percentage increased to 16%, 25%, 44%, and 47% for 10%, 15%, 20%, and 25% CKD replacement, respectively.

## 5. Assessment of the Application Tests

Table 8 shows the statistical results of the various tests: mean values for the ratio of each parameter (concrete compression and tensile strength, air content, and block compressive strength) and the CKD percentage along with the standard deviation and the minimum and maximum values.

It can be seen that having more than 5% CKD had a significant impact on the sample properties. Therefore, it is recommended that a replacement of 10% CKD can be used with an acceptable reduction in concrete and block strength.

## 6. Summary and Conclusions

The effects of CKD in plain concrete on compressive strength, tensile strength, and air content were studied, while the effects of CKD in cement blocks on compressive strength and water absorption were investigated. The experiments revealed the following:

- CKD has a detrimental impact on compressive strength, as evidenced by the fact that as the amount increased, the compressive strength of the concrete and cement block specimens decreased. An equation corresponding to each was presented to anticipate this reduction.
- Regarding the concrete tensile strength, there was no significant difference between 0% and 5% CKD, which was only approximately 3%.
- In the concrete mix, the percentage of air content from 0% to 5% CKD replacements made no difference; however, when CKD was increased to 10%, 15%, and 20%, it increased by 24%, 33%, and 43%, respectively.
- The percentage of water absorption due to partial replacement by CKD in cement blocks can be increased up to 25% within the allowed limits.
- This research demonstrates that CKD can be used as a primary component in concrete (e.g., plain concrete, curbs, and cement tiles) and cement block products.

## Figures and Tables

**Table 1:** List of tests and specimens conducted.

No.	Test	Specimen Dimension	No. of Specimens
1	Chemical analysis	Cement kiln dust (CKD)	1
2	Concrete compressive strength	Cube (150 mm × 150 mm × 150 mm)	42
3	Concrete tensile strength	Cylinder (15 mm in diameter; 30 mm in height)	12
4	Concrete air content	Gilson container	15
5	Blocks compressive strength	Hollow cement blocks (200 mm × 200 mm × 400 mm)	30
6	Block absorption	Hollow cement blocks (200 mm × 200 mm × 400 mm)	20

**Table 2:** Typical chemical compositions of CKD and ordinary portland cement (OPC) in the UK and KSA.

Chemical Composition (%)	CKD (UK) [14]	CKD AL Madina Cement Factory (AL Madina KSA)	OPC
SiO_2_	11–16	18.20	22
Al_2_O_3_	3–6	4.52	5
Fe_2_O_3_	1–4	2.92	3
CaO	38–50	49.40	64
MgO	0–2	1.21	1
SO_3_	4–18	5.66	3
K_2_O	3–13	2.38	<1
Na_2_O	0–2	3.84	<1
Cl	0–5	5.90	<0.10
Loss on ignition	5–25	17.10	1
Free Cao	1–10	4.24	2

**Table 3:** Concrete sample compressive strength results.

No.	Cube Compressive Strength after 28 Days (MPa)							
	% of CKD	0%	2%	5%	8%	10%	15%	20%
1	From cement portion in concrete mixes	29	28.2	28	27.6	27	24.5	19.2
2		29.3	29	27.5	27.3	26.3	25	18.5
3		28.45	28.1	27.6	26.5	26.5	23.8	18.2
4		28.41	28.3	28	27	26.8	23.7	19
5		28.66	28	27.5	26.4	26.2	24.2	18.4
6		28.5	28.1	28.2	27.2	26.2	24	18.3
Average (MPa)		28.724	28.45	27.8	27	26.5	24.2	18.6

**Table 4:** Concrete tensile strength results.

No	% of CKD	Tensile Strength (MPa)						Average (MPa)
		1	2	3	4	5	6	
1	0%	2.97	3.11	2.83	2.90	3.00	2.95	2.95
2	5%	2.81	2.86	2.68	2.74	2.85	2.87	2.80

**Table 5:** Concrete air content results.

No. (1)	% CKD (2)	% Air Content (3)	% Air Content Average (4)
1	0	1.05	1.05
2		1.05	
3		1.00	
1	5	1.05	1.05
2		1.05	
3		1.10	
1	10	1.30	1.30
2		1.35	
3		1.25	
1	15	1.35	1.40
2		1.45	
3		1.35	
1	20	1.45	1.50
2		1.60	
3		1.50	

**Table 6:** Hollow block compressive strength results.

% of CKD	Crushing Load (kN)	Strength (MPa) Net Area		Strength (MPa) Gross Area	
		Per Specimen	Average	Per Specimen	Average
0	55.5	12.31	11.89	7.30	7.05
	56.00	12.42		7.36	
	52.30	11.60		6.88	
	50.50	11.20		6.64	
	52.75	11.70		6.94	
	54.56	12.10		7.18	
10	48.25	10.70	10.50	6.34	6.22
	47.80	10.60		6.29	
	45.70	10.14		6.01	
	47.50	10.54		6.25	
	48.00	10.65		6.32	
	46.64	10.35		6.14	
15	37.82	8.39	9.50	4.97	5.62
	46.7	10.36		6.14	
	42.2	9.36		5.55	
	44.3	9.83		5.83	
	43.17	9.58		5.68	
	42.59	9.45		5.60	
20	35.11	7.79	7.80	4.62	4.63
	35.98	7.98		4.73	
	34.24	7.6		4.50	
	35.41	7.85		4.66	
	35.27	7.82		4.64	
	35.00	7.76		4.61	
25	27.34	6.06	6.15	3.60	3.65
	28.2	6.26		3.71	
	27.56	6.11		3.62	
	27.82	6.17		3.66	
	27.73	6.15		3.65	
	27.59	6.12		3.63	

**Table 7:** Hollow blocks absorption results.

% CKD	Weight (*N*)			Average Weight (*N*)		% Absorption
	After Oven	After Submerged	Water Absorbed	Dry Weight	Water	
**0**	196.35	204.20	7.85	191.64	8.18	4.27
	189.70	198.15	8.45			
	191.00	198.90	7.90			
	189.50	198.00	8.50			
10	196.80	205.90	9.10	190.13	9.48	4.98
	186.20	196.10	9.90			
	186.50	196.40	9.90			
	191.00	200.00	9.00			
15	185.75	195.95	10.20	185.61	9.913	5.34
	185.40	195.15	9.75			
	185.20	195.00	9.80			
	186.10	196.00	9.90			
20	186.75	197.95	11.20	187.01	11.53	6.16
	187.60	199.40	11.80			
	186.60	198.20	11.60			
	187.10	198.60	11.50			
25	182.45	194.20	11.75	186.04	11.74	6.31
	188.00	199.80	11.80			
	188.30	200.00	11.70			
	185.40	197.10	11.70			

**Table 8:** Assessment of various test specimens.

Test Type	% CKD	Mean	Standard Deviation	Min.	Max
Concrete compressive strength	2	0.985	0.009	0.972	0.996
	5	0.968	0.019	0.939	0.989
	8	0.940	0.014	0.921	0.954
	10	0.923	0.016	0.898	0.943
	15	0.843	0.007	0.834	0.853
	20	0.648	0.014	0.631	0.669
Concrete tensile	5	0.950	0.017	0.920	0.973
Concrete air content	5	1.033	0.058	1.000	1.100
	10	1.258	0.025	1.238	1.286
	15	1.339	0.049	1.286	1.381
	20	1.468	0.077	1.381	1.524
Block compressive strength	10	0.884	0.019	0.862	0.905
	15	0.798	0.028	0.749	0.834
	20	0.657	0.017	0.638	0.679
	25	0.517	0.016	0.501	0.541

## Data Availability

The data presented in this study are available on request from the corresponding author.
